# Attention-Guided Multi-Scale Feature Fusion Network for Low-Light Image Enhancement

**DOI:** 10.3389/fnbot.2022.837208

**Published:** 2022-03-03

**Authors:** HengShuai Cui, Jinjiang Li, Zhen Hua, Linwei Fan

**Affiliations:** ^1^College of Electronic and Communications Engineering, Shandong Technology and Business University, Yantai, China; ^2^Institute of Network Technology, Institute of Computing Technology (ICT), Yantai, China; ^3^School of Computer Science and Technology, Shandong University of Finance and Economics, Jinan, China

**Keywords:** low-light image enhancement, multi-scale, attention mechanism, feature calibration, cascade fusion, coarse-to-fine

## Abstract

Low-light image enhancement has been an important research branch in the field of computer vision. Low-light images are characterized by poor visibility, high noise and low contrast. To improve low-light images generated in low-light environments and night conditions, we propose an Attention-Guided Multi-scale feature fusion network (MSFFNet) for low-light image enhancement for enhancing the contrast and brightness of low-light images. First, to avoid the high cost computation arising from the stacking of multiple sub-networks, our network uses a single encoder and decoder for multi-scale input and output images. Multi-scale input images can make up for the lack of pixel information and loss of feature map information caused by a single input image. The multi-scale output image can effectively monitor the error loss in the image reconstruction process. Second, the Convolutional Block Attention Module (CBAM) is introduced in the encoder part to effectively suppress the noise and color difference generated during feature extraction and further guide the network to refine the color features. Feature calibration module (FCM) is introduced in the decoder section to enhance the mapping expression between channels. Attention fusion module (AFM) is also added to capture contextual information, which is more conducive to recovering image detail information. Last, the cascade fusion module (CFM) is introduced to effectively combine the feature map information under different perceptual fields. Sufficient qualitative and quantitative experiments have been conducted on a variety of publicly available datasets, and the proposed MSFFNet outperforms other low-light enhancement methods in terms of visual effects and metric scores.

## 1. Introduction

Nowadays, more and more researchers are working to solve the problem of image degradation in poorly illuminated scenes. Low-light image enhancement methods aim to restore image sharpness and contrast, as well as detailed information in dark-light regions, which is a very challenging task. In low-light environments, due to the limitations of image acquisition equipment, the photographs taken often have low brightness, low contrast and severe noise phenomena. Although the use of expensive photography equipment and the involvement of professionals can reduce image degradation to a certain extent, the photos still have overexposure and blurred objects. Low-light images not only affect the user's visual perception, but also seriously affect the processing of advanced computer vision tasks (target detection and recognition). Low-light image enhancement is closely related to various high-level vision tasks, and the enhanced low-light images can bring more hidden information. Therefore, low-light image enhancement technology is essential to restore low-light images to low-noise, high-contrast, and high-quality images with normal colors. The low-light enhanced image can provide good preconditions for the subsequent target detection (Lin et al., 2017; Shen et al., 2020; Wang et al., 2020b), image recognition (Shi et al., 2020; Zhao et al., 2020),image segmentation (Zhang et al., 2021), image classification (Liu et al., 2020b) and autonomous driving (Chen et al., 2016; Prakash et al., 2021) and other advanced vision tasks. Meanwhile, visual information processing is also inseparable in fields such as military missions and deep-sea environments and Biomedical imaging (Ardizzone et al., 2006, 2008).

Early conventional low-light enhancement methods were applied to degraded images with low brightness, low contrast and artifacts. The histogram equalization (HE) (Pisano et al., 1998; Abdullah-Al-Wadud et al., 2007) method counts the frequency of each pixel value to adjust the gray value difference between pixels, and obtains an enhanced image with uniform gray value distribution through transformation. The method based on Retinex (Land, 1977) theory decomposes the image into illumination and reflection layers. The single-scale Retinex (SSR) (Jobson et al., 1997b) algorithm and the multi-scale Retinex (MSR) (Jobson et al., 1997a) algorithm have been proposed successively. Later the Multi-Scale Retinex for Color Recovery (MSRCR) (Jiang et al., 2015) method added color balance processing to the former method. Although the contrast and brightness of low-light images are improved, the enhanced low-light images still have insufficient edge sharpening and weak color retention. Because the classic Retinex method assumes that the light is located in the low-frequency component, but the halo phenomenon will occur in the area with large differences in brightness and the image will be distorted. Wang et al. (2019c) introduced the intermediate illumination component, and used the loss function to constrain and a priori the illumination component. Due to the lack of noise removal in this method, the visual effect is unsatisfactory. Fu et al. (2016) proposed a weighted variational model and estimated illumination and reflection to obtain prior representation. The enhanced image can retain more information details and suppress the generation of noise. Although these methods enhance the contrast of low illumination images, the enhanced images suffer from strong noise and lack of details.

**Figure 1:**
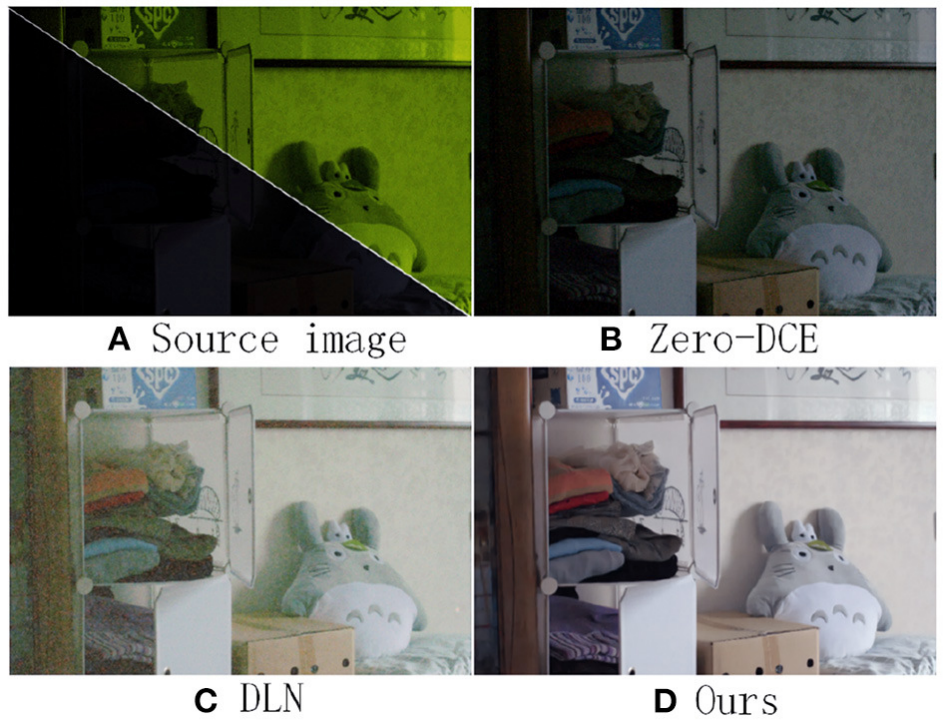
**(A)** shows the original low-light image; **(B)** the result after enhancement by Zero-DCE method; **(C)** the result after enhancement by DLN method; **(D)** the result after enhancement by our method. **(A)** is poorly lighted, so the image is shown in the upper right corner after zooming in on the infrared and adding visible light.

In recent years, with the improvement of computer computing performance, a series of low-light enhancement methods based on neural network algorithms have achieved good results. Figure 1A shows the source low light image in a low light scene. (Lore et al., 2017) and (Wei et al., 2018) proposed LLNet and Retinex-NET network models respectively, which solved the problems of difficult decomposition model and complex parameter design of traditional retinex methods. (Zhang et al., 2019) decoupled the image into two parts and added a denoising network for removing the degradation in the dark region after amplification. the Zero-DCE(Guo et al., 2020) method of Figure 1B uses an unsupervised approach for low-light enhancement. The unsupervised Zero-DCE method used lacks validity guidance, and the enhanced images suffer from underexposure and poor color recovery. In addition, (Wang et al., 2020a) proposed a Deep Lightening Network (DLN), which treats low-light enhancement tasks as residual tasks and uses super resolution back projection technology for lowlight enhancement. From Figure 1C is the image after enhancement by the DLN method, which results in a large amount of noise and loss of texture details. Figure 1D is the image enhanced by our MSFFNet method, which has the characteristics of low noise and high brightness. In general, the difficulty of low-light image enhancement tasks lies in recovering dark information and suppressing noise generation. The above proposed method is still a challenging task for simultaneous contrast enhancement, noise removal, and color recovery.

In this paper, we propose an Attention-Guided Multi-scale feature fusion network for low light image enhancement based on the structure of a single encoder and decoder and the principle of coarse-to-fine network design. The novelty of our proposed method is summarized in the following aspects:

1. The framework uses a multi-scale input and output network architecture, where low-light images at different scales are input into the encoder for extracting shallow and deep feature information at different scales. Adding lightweight Convolutional Block Attention Module (CBAM) to each encoder block enables the network to emphasize important feature information under the channel and space and recover the hidden detail content.
2. The output of enhanced images at different scales in the decoder facilitates the network to be able to reconstruct image information from coarse to fine. At the same time, in order to reduce the loss of details caused by the deepening of network layers, a feature calibration module (FCM) is introduced to adjust the weight distribution of features and enhance the expression ability of the network. In order to capture global context information, a cross-level fusion attention fusion module (AFM) is added to each encoder sub-block to further enhance the effect of low-light images.
3. A cascade fusion module (CFM) is designed to fuse multi-scale information to improve the information flow between networks and enhance the ability of global and local feature information fusion.

## 2. Related work

Low-light image enhancement has been a widely researched direction in the field of computer vision. The existing low-light enhancement methods can be broadly classified into three types: histogram equalization-based methods, Retinex-based methods, and deep learning-based methods. We will briefly introduce the most relevant methods for low-light image enhancement and the application of attention mechanisms.

### 2.1. Histogram Equalization-Based Methods

The HE method is the simplest and most straightforward method for low-light enhancement. The histogram equalization method stretches the image non-linearly, so that each gray value is evenly distributed, and the image contrast is improved. Adaptive histogram equalization (AHE) (Pizer et al., 1987) is used to enhance the image contrast, but the enhanced image has a lot of noise. Wang and Ng (2013) proposed a variational approach to consider local information around pixels and perform local transformations to enhance image contrast. The brightness preserving dynamic histogram equalization (BPDHE) (Ibrahim and Kong, 2007) method uses Gaussian filters to partition the dynamic range and change the average brightness of the image. The CVC (Tsai et al., 2012) method searches for the dependency between each pixel and the corresponding neighborhood, and makes full use of the contextual information between pixels to enhance the contrast. Lee et al. (2013) proposed an algorithm based on two-dimensional histogram to represent gray differen-ce in a hierarchical manner, which changed the image contrast by increasing the gray difference between each adjacent pixel. Although these methods only dynamically adjust the range of gray levels and improve the contrast of the image, the enhanced image will have problems such as uneven color, amplified noise, and loss of detail.

### 2.2. Retinex-Based Methods

Inspired by the theory of Retinex (Land, 1977), any images can be expressed as the product of the illumination component and the reflection component. In the earlier SSR (Jobson et al., 1997b) method and MSR (Jobson et al., 1997a) method, illumination is obtained from various a prior information and adaptively adjusted for image enhancement. The enhanced low-light image has the phenomenon of distorted color and insufficient edge sharpening. Wang et al. (2013) proposed an enhancement algorithm for non-uniformly illuminated images to maintain detail and natural balance. Ren et al. (2018) proposed a continuous sequence performing Retinex decomposition for low-light image enhancement and denoising. Hao et al. (2020) used a semi-decoupled approach to effectively implement image decomposition to improve the visibility and visual quality of low-light images. Guo et al. (2016) performed low-light image enhancement by finding the maximum pixel value in the three RGB channels for estimating the illumination of each pixel and adding a priori structure to optimize the illumination map. Li et al. (2018) first considered the noise problem after decomposing images and proposed a robust Retinex model for optimizing the structural details of low-light images. Although the method based on Retinex theory aims to accurately estimate the illumination components, the estimated illumination components still have errors due to the complexity of image decomposition and the nonlinearity of the channels. As a result, the enhanced low-light image still suffers from detail loss and color distortion.

### 2.3. Deep Learning-Based Methods

In recent years, deep learning has achieved good results in the areas of image dehazing (Dong et al., 2020), image denoising (Zhang et al., 2017), image super-resolution (Tai et al., 2017) and image detection (Li et al., 2020a). Researche-rs have also successively proposed a series of methods for low-light image enhancement based on deep learning. These methods can be divided into those based on convolutional neural networks and those based on generative adversarial networks.

The CNNs-based methods require paired datasets for training. Lore et al. (2017) designed low-light image enhancement networks for contrast enhancement and denoising using stacked sparse denoising autoencoders. Li et al. (2021) proposed a recursive unit composed of recurrent and residual layers for progressive low-light image enhancement. Jobson et al. (1997a) proposed an enhancement network that uses multiple sub-networks to extract features at different levels for multi-branch fusion to achieve mapping from low-light images to enhanced images. Wei et al. (2018) proposed an enhanced network integrated with decomposition network and illumination adjustment, called Retinex-NET. In joint denoising, existing denoising tools (BM3D Dabov et al., 2007) are used. The enhancement network is used to adjust the light component to achieve end-to-end low light enhancement. Wang et al. (2019a) proposed a new Lightening Back-Projection (LBP) block, which iteratively learns the differences between low-light images and normal light images for low-light enhancement. Although these methods restore the feature information of low brightness image to a certain extent, the enhanced image still has strong noise and color imbalance.

In addition to CNNs methods, there are GANs-based methods that are also widely used for low-light image enhancement. Chen et al. (2018) proposed an improved bidirectional GANs learning method to achieve mapping from low-light images to normal-light images. The lack of considering edge information leads to blurred edge sharpening of the enhanced image. Jiang et al. (2021) designed an unsupervised generative adversarial network to normalize the feature information of unpaired low-light images. Guo et al. (2020) proposed a lightweight depth network to estimate dynamic range-adjusted pixels and curves. The network can be applied to low-light images under different light intensities by nonlinear curve mapping. Due to the lack of reference data as constraints in the GANs-based network model, the enhanced low-light image still has underexposure and strong noise.

### 2.4. Attention Mechanism

The attention mechanism has become an important part of neural networks and is widely used in fields such as natural language processing, speech recognition, and computer vision. The attention module enhances network expression ability by emphasizing useful features and suppressing useless feature information. In the field of computer vision, Hu et al. (2018) proposed the Squeeze-and-Excitation module to study the dependencies between channels and to assign weights to different channels by the learned weight information. Later, Woo et al. (2018) proposed the Convolutional Block Attention Module (CBAM) to increase attention in two dimensions, channel and space, respectively, to refine feature information and guide the network to focus on important content. To establish the connection between two pixels, Wang et al. (2018) proposed a non-local operation to capture the dependency between two locations. To improve model training performance and reduce computational complexity, Wang et al. (2019b) proposed the Efficient Channel Attention (ECA) module to perform cross-channel interaction and keep the performance constant. Liu et al. (2020a) proposed a residual feature aggregation (RFA) network for image super-resolution. The framework introduces a lightweight and efficient enhanced spatial attention (ESA) module, which makes the residual feature information more focused on the spatial content, reduces the complexity of the model, and improves the image visualization. Therefore, the introduction of the attention mechanism can assign important weights to different feature information and suppress non-important information. In other words, the mechanism can effectively suppress unnecessary color features and noise, and adaptively enhance the useful information of features.

## 3. Proposed method

**Figure 2:**
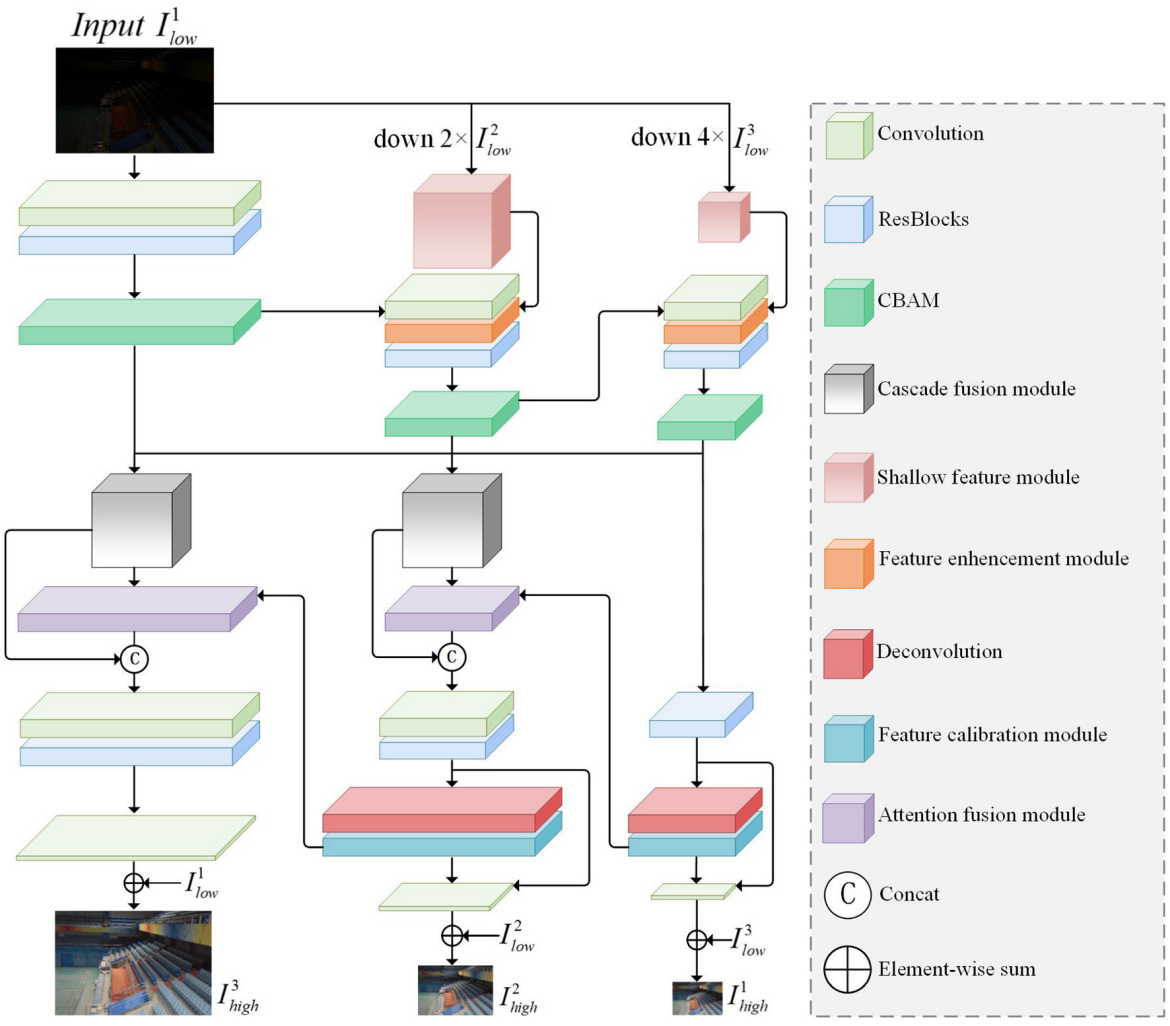
The overall framework of our proposed MSSFNet. The network framework is divided into two parts: the upper part is the encoder part for multi-scale input images and the lower part is the decoder part for multi-scale output images.

As shown in Figure 2, we designed an Attention-Guided Multi-scale feature fusion network(MSFFNet) for low-light image enhanc-ement. By combining the output multiscale images to calculate losses to guide the training process, the multiscale features of low-light images are fully utilized, and the loss of texture details in the image recovery process is greatly avoided. Different from the original U-NET (Weng and Zhu, 2015), in order to better adapt to the task of multi-scale low-light enhanced image, we input multi-scale image to each encoder block and output multi-scale image to the decoder block of each layer. In this section, we will detail the overall structure of the network and the various functional modules of the network, as well as the loss function.

### 3.1. Multi-Scale Input Encoder and Output Decoder

The existing CNN network structure is usually based on the encoder and decoder for low-light image enhancement, and has shown excellent performance. Low-light image enhancement with a single input image will cause the loss of detail and texture when restoring the dark-light area. In order to be able to better deal with different degrees of information loss in images, we use low-light images of different scales as the input to each sub-network. It has been demonstrated that multiscale images have good enhancement effects in image deblurring (Cho et al., 2021; Zhang et al., 2022), image derainting (Jiang et al., 2020), and image dehazing (Li et al., 2020b).

Different from stacked sub-networks, the overall part of our network architecture consists of a single encoder and decoder. The encoder and decoder are composed of three encoder blocks (ENCs) and three decoder blocks (DECs). The original encoder and decoder extract image features from a single image in order to restore the hidden information of the image. Although the receptive field is enlarged in the down-sampling process, there is often the loss of characteristic information. Therefore, in the encoder part, in addition to extracting information in a single encoder block, we also input low-light images at different scales in each ENC for feature fusion of the downsampled feature information and the shallow feature information extracted at low scales, which can reduce the information loss after image downsampling. In the decoder part, each DEC block outputs the reconstructed feature maps of different sizes separately for the calculation of the loss function.

**Figure 3:**
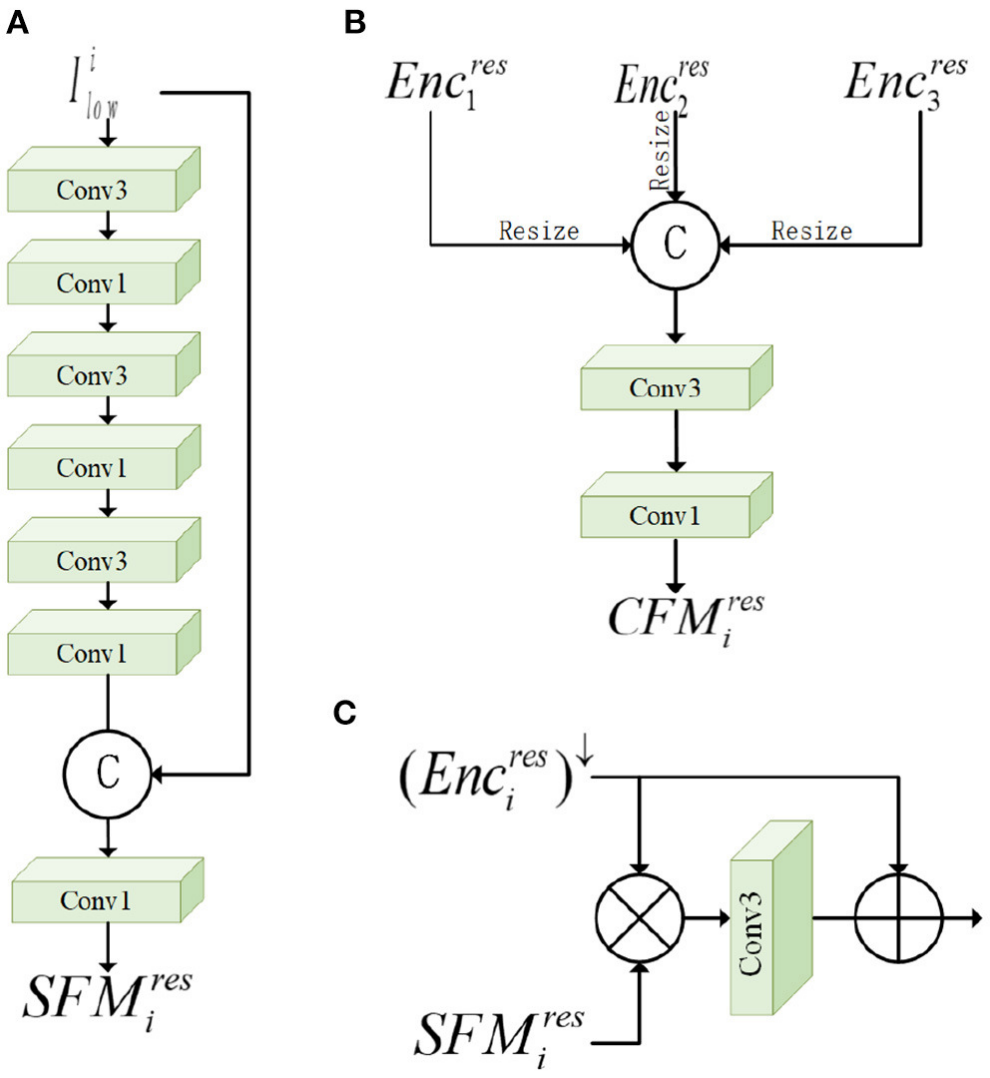
The various sub-modules of the network: **(A)** Shallow feature module (SFM), **(B)** cascade fusion module (CFM), and **(C)** feature enhancement module (FEM).

Firstly, for the input low-light image, we use the nearest neighbor interpolation (nearest) method to downsample twice consecutively to generate 1/2 and 1/4 of the original low-light image for use in ENCs. For low-light images at different scales, we use the shallow feature module (SFM) to perform shallow feature extraction on the downsampled images, as shown in Figure 3A. SFM performs convolutional operations on the image by three successive 3 × 3 and 1 × 1 convolutional layers. Then we connect the input image $I_{\text{low}}^{i}$ and the features after shallow convolution, and further refine the fused feature information by a 1 × 1 convolution layer. The low-light image after SFM is denoted as $SFM_{i}^{res}$, where *i* denotes the kth layer and res denotes the result after output. In the whole network framework, we add SFM for shallow feature extraction in the initial stage of the second and third layers, as shown in Figure 2. To fuse $SFM_{i}^{res}$ with the feature information $ENC_{i-1}^{res}$ from the previous layer, we pass $ENC_{i-1}^{res}$ through a convolutional layer with a step size of 2 and a convolutional kernel of 3 × 3 to generate ${\left(ENC_{i-1}^{res}\right)}^{↓}$ of the same size as $SFM_{i}^{res}$. Next, the feature enhancement module (FEM) is used, as shown in Figure 3C. FEM effectively combines two features $SFM_{i}^{res}$ and ${\left(ENC_{i-1}^{res}\right)}^{↓}$. It is able to reinforce multi-scale contextual key feature information and suppress non-key feature information, effectively utilizing the shallow spatial and channel information learned from SFM. Specifically, the individual pixels of $SFM_{i}^{res}$ and ${\left(ENC_{i-1}^{res}\right)}^{↓}$ are multiplied, and then the hidden feature information is obtained through a 3*3 convolution layer, and finally this feature information and ${\left(ENC_{i-1}^{res}\right)}^{↓}$ are summed for each element to obtain a feature map containing the hidden information. With the increase of network depth, degradation may occur. Adding residual blocks can effectively solve the gradient dispersion and network degradation and maintain the performance of the network. Finally, we added the attention module CBAM to guide the enhancement of under-exposed areas and avoid over-enhancement of normal highlighted information, which will be detailed in Section 3.2.

In the multiscale decoder part, the DEC blocks of each layer output feature maps of the corresponding size, which produce image artifacts due to a tessellation effect after deconvolution. In order to eliminate this phenomenon, a feature calibration module (FCM) is added after deconvolution to capture spatial correlation. The feature maps correspon-ding to different channels have different attention features. In order to fully combine the important feature information at different scales, the attention fusion module (AFM) is introduced to generate more discriminative feature representation. At the same time, we connect the feature information received from the input images at each level to the current DEC block, and the image reconstruction features obtained at each level can be expressed as:


(1) $$\begin{array}{l} I_{high}^{k}=\{\begin{array}{c} F\left(DEC_{k}\left(\alpha ;\beta \right)\right)+I_{low}^{k},k=1,2\\ F\left(DEC_{k}\left(ENC_{k}^{res}\right)\right)+I_{low}^{k},k=3 \end{array}, \end{array}$$

where $DEC_{k}^{res}$, $ENC_{k}^{res}$ are the results after the output of the DEC block, ENC block at the *k*^*th*^ level, respectively. α and β are denoted as $CFM_{k}^{res}$ and $DEC_{k+1}^{res}$, respectively, and *DEC*_*k*_(;) denotes the concatenation operation performed at the kth level. Since the output after DEC block is the feature map, convolution operation by mapping function *F*(·) is used to recover the reconstructed image after each layer.

### 3.2. Attention Mechanism

#### 3.2.1. Convolutional Block Attention Module

In order to be able to properly guide the network to focus on important feature information and improve the learning ability of the neural network. CBAM (Woo et al., 2018) was introduced in the network for adaptive feature refinement. The attention mechanism effectively calculates the channel attention and spatial attention of the feature map, which can capture the hidden information more accurately, recover the dark region information and suppress the generation of noise. The module computes the attention maps from two dimensions, spatial and channel, respectively, and the resulting attention maps and the original input feature maps are multiplied sequentially.

Firstly, the intermediate feature map with a size of C × W × H was taken as the input of CBAM, and important channel information was obtained by compressing spatial dimensions in the channel attention module. Then, the spatial attention module is further supplemented to make the network precisely focus on low-light hidden information. Specific operations can be expressed as follows:


(2) $$\begin{array}{l} F_{c}=M_{c}^{i}\left(F_{in}\right)⊗F_{in},i=1,2,3\\ F_{s}=M_{s}^{i}\left(F_{c}\right)⊗F_{c},i=1,2,3, \end{array}$$

where ⊗ represents element-wise multiplication, the intermediate feature map is represented as *F*_*in*_,$M_{c}^{i}$ and $M_{s}^{i}$ represent the attention maps of the *i*^*th*^ layer through channel and space mapping. *F*_*c*_ represents the global feature information refined by the channel attention, and *F*_*s*_ represents the local feature information further refined by the spatial attention.

#### 3.2.2. Attention Fusion Module

**Figure 4:**
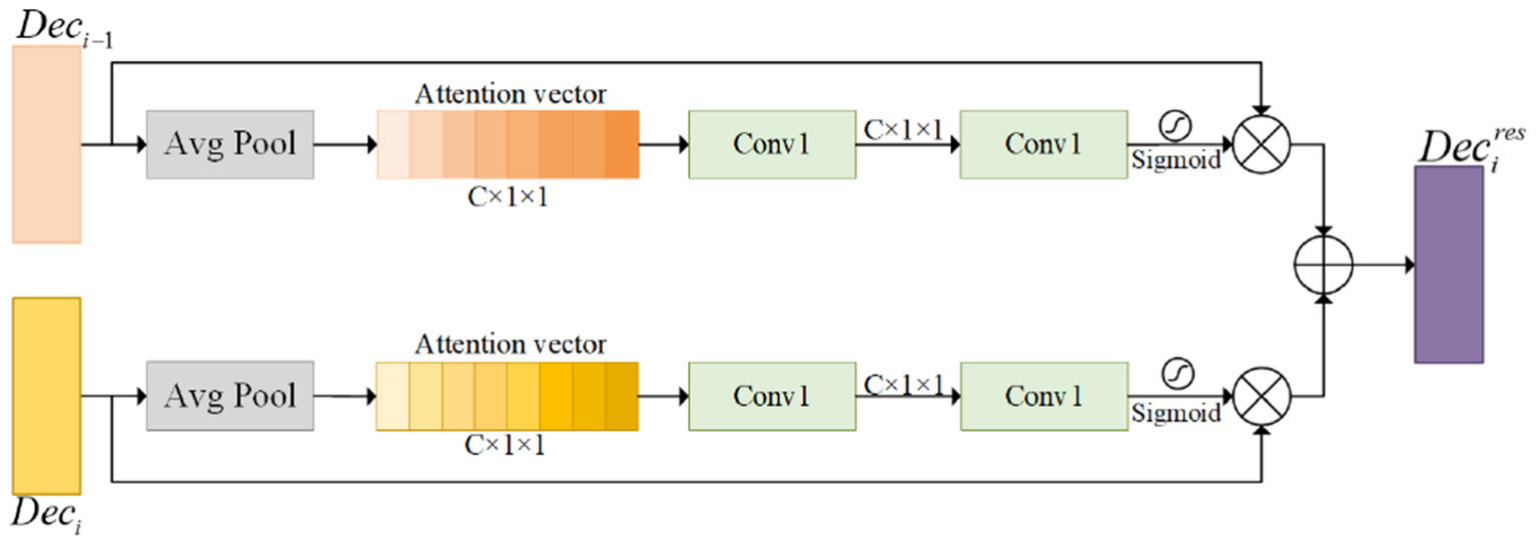
This module is the Attention Fusion Module (AFM). The ⊗ represents element-wise multiplication, and the ⊕ represents element-wise sum.

In order to effectively fuse two feature maps with different information contents while being able to focus on key target regions, an attention fusion module (AFM) is introduced to fuse the attention of the two feature maps, effectively complementing the information of different levels of feature map contents. This module enhances the distinguishability of features by adjusting the weights of each channel feature. Inspired by the squeeze-and-excitation mechanism (Hu et al., 2018), our proposed AFM recodes the semantic correlation between channels for the multi-scale fused feature maps and the low-level feature maps, respectively. The AFM extracts optimized weight information for both features in parallel, focusing attention on the target region, as shown in Figure 4. The important target weight information is obtained in the low-level feature map to emphasize the hidden details. The dynamic channel features will be further calibrated by multi-scale fusion information from the cascade fusion module (CFM), which helps to capture global contextual information more accurately and reinforce important channel information.

The AFM takes the low-level and multi-scale fused feature maps and enhances semantic dependencies and boosts channel relevance through global averaging pooling operations, respectively. The global features are compressed into an attention vector to facilitate the exploitation of contextual information beyond the local sensory field. To exploit the information in the attention vector, a convo-lutional transformation is then performed to further capture the channel dependencies. The specific operation of generating the attention vector is represented as follows:


(3) $$\begin{array}{l} F_{Att}=\lambda_{1}\left[W_{1}\cdot \lambda_{2}\left[W_{2}\cdot GAP\left(x\right)+b_{1}\right]+b_{2}\right], \end{array}$$

where *x* denotes the input feature map. GAP represents the global average pooling operation, and the mathematical formula is as follows. λ_1_ and λ_2_ denote ReLU activation functions and Sigmoid activation functions, respectively. *W*_1_ and *W*_2_ denote convolution kernels for 1 × 1 convolution operations. *b*_1_ and *b*_2_ are biases.


(4) $$\begin{array}{l} GAP\left(x_{k}\right)=\frac{1}{W\times H}\overset{H}{\underset{i=1}{\sum }}\overset{W}{\underset{j=1}{\sum }}x_{k}\left(i,j\right) \end{array}$$

where W and H denote the width and length of the feature map. *k* = 1, 2, …, *c*, c is the number of channels of the feature map. *x* = [*x*_1_, *x*_2_, …, *x*_*c*_].


(5) $$\begin{array}{l} x_{res}=F_{Att}⊗x, \end{array}$$

where ⊗ represents the element-by-element multiplication operation. *x*_*res*_ is the final output feature map result.

Finally, the two feature maps recalibrated by the attention module are additively merged. Compared with the Concatenate operation, the addition operation reduces the parameters of the convolution and avoids the increase of computational cost. The final result obtained is the feature map after enhanced attention.

### 3.3. Cascade Fusion Module

Since the network is accompanied by informat-ion loss during downsampling, we fuse each encoder block extracting informat-ion at different scales for removing dark information to reproduce hidden texture detail features. CFM designed by us is introduced into the network, which is beneficial to supplement the feature map after loss and enables cross-scale communication and enhanced information flow between different layers of the network. The module is shown in Figure 3B. This module cascades the whole vertical direction of the high-level semantic information and low-level semantic information in different scales, enriches the multi-scale feature map, and improves the training accuracy of the network. Each CFM fuses the output information of three DECs blocks and further refine the multi-scale features using two-level convolution. The cascaded fusion information is passed into the specified ENCs, and the CFM output results corresponding to the first and second layers are expressed as *CFM*_1_ and *CFM*_2_. The formula is expressed as follows:


(6) $$\begin{array}{l} CFM_{1}^{res}=CFM_{1}\left(ENC_{1}^{res},{\left(ENC_{2}^{res}\right)}^{↑},{\left(ENC_{3}^{res}\right)}^{↑}\right)\\ CFM_{2}^{res}=CFM_{2}\left({\left(ENC_{1}^{res}\right)}^{↓},ENC_{2}^{res},{\left(ENC_{3}^{res}\right)}^{↑}\right), \end{array}$$

where $CFM_{i}^{res}$ represents the multi-scale feature fusion result output by the *i*^*th*^ layer. Up-sampling and down-sampling operations are denoted by ↑ and ↓, respectively, to facilitate the connection of low-light image feature information at multiple scales. We fuse feature information at different scales to reduce the loss of target information and help restore image content and detailed information.

### 3.4. Feature Calibration Module

**Figure 5:**
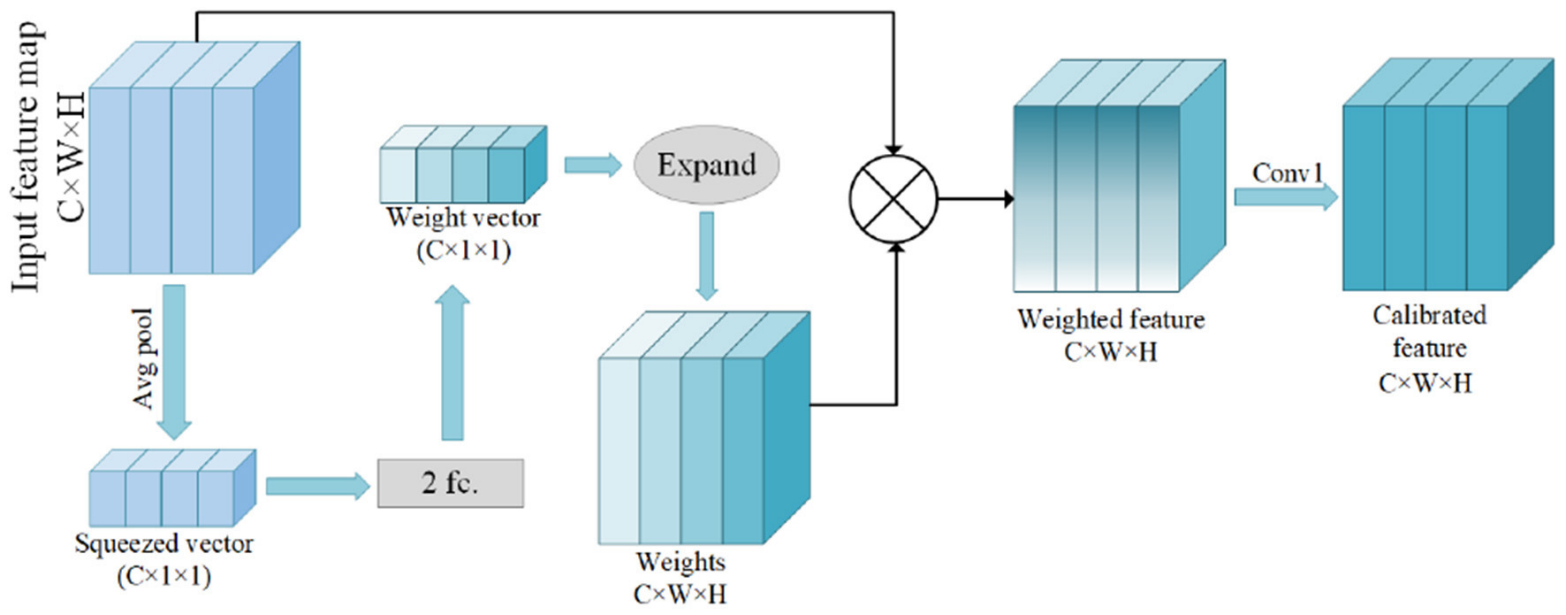
This module is the Feature Calibration Module (FCM).

In order to improve image degradation to enhance detail clarity and restore good visual quality, FCM is introduced for low-light enhancement of global and local features, as shown in Figure 5. During the processing of the low-light enhancement task, the global information is used to evaluate the low-light image lighting conditions, and the local feature information is enhanced to optimize the detail content to enhance the feature expression. Due to the deepening of the network layers, the perceptual field expansion also increases the risk of gradient disappearance and gradient explosion, for which we add residual blocks before deconvolution to optimize the performance of the network. Then, we input the feature map of size W × H and number of channels C to FCM for feature weight recalibration after deconvolution.

Inspired by the adaptive calibration of channel weights (Hu et al., 2018) to improve the sensitivity of channel information, each channel information is first compressed through the global average pooling layer to obtain the dependencies between each other, and the feature map is compressed into a C × 1 × 1 compressed vector. The values of the compressed vectors represent the information of the channels. The channel correlations are investiga-ted by using two fully connected layers, each with activation functions of ReLU and Sigmoid functions, respectively, to estimate a weight vector of size C × 1 × 1. Then, the weight vectors are expanded in width and height, respectively, and the recovered dimension size is C × W × H. The feature maps and the corresponding weights are multiplied element by element to obtain the calibrated feature maps.

The recalibrated key information may have larger weights, causing the key information to be too prominent. For this reason, we optimize the expressiveness of the weighted feature maps by convolutional layers with 1 × 1 convolutional kernels, and finally obtain the weight-calibrated feature maps, which are beneficial to the subsequent calculations.

### 3.5. Loss Function

The loss function plays a key role in the performance of the model. For multi-scale low-light enhancement networks, the content information during the multi-scale reconstruction of images can be fully utilized. Therefore, we calculate the loss for each pair of pixels between the reconstructed image and the corresponding real reference image output in each decoder layer. The content accuracy after reconstructing the image is calculated using L1 loss (MAE loss). Inspired by Li et al. (2020b), we accumulate the content loss of the images after low-light enhancement at different scales, which is beneficial to monitor the error loss of the images at different scales. The content loss is calculated as follows:


(7) $$\begin{array}{l} Loss_{con}=\overset{N}{\underset{i=1}{\sum }}\frac{1}{x_{i}}{\left\|I_{high}^{i}-I_{gt}^{i}\right\|}_{1}, \end{array}$$

where N denotes the number of layers in the network. The content loss function is normalized by dividing it by *x*_*i*_ (the total number of pixels). $I_{i}^{high}$ and $I_{i}^{gt}$ are the low light enhancement image and ground true image during the reconstruction process, respectively.

In order to be able to reduce the loss of details and edge features, we add auxiliary losses to recover the lost high frequency components. A large amount of noise occurs in the image reconstruction process, and the noise mainly exists in the high frequency part. In order to reduce the discrepancy in the frequency domain space, we propose a multi-scale frequency domain (MSFD) loss function applied in the loss calculation. The L1 loss expression between the multiscale ground truth image and the low light enhancement image is calculated in the frequency domain as follows:


(8) $$\begin{array}{l} Loss_{MSFD}=\overset{N}{\underset{i=1}{\sum }}\frac{1}{x_{i}}{\left\|f\left(I_{high}^{i}\right)-f\left(I_{gt}^{i}\right)\right\|}_{1}, \end{array}$$

where *f* denotes the operation of transforming the image signal into the frequency domain by the fast Fourier transform.

Finally, the mixed loss function defined by us is expressed as follows:


(9) $$\begin{array}{l} Loss_{total}=Loss_{con}+\lambda Loss_{MSFD}, \end{array}$$

where λ is a balance parameter, set to 0.1. The model uses *Loss*_*total*_ loss function to train end-to-end to network convergence.

## 4. Experiment

In this section, we detail the experimental details to demonstrate the validity of the method. Firstly, we present the data set and experimental details as well as the evaluation metrics. Secondly, we perform a qualitative and quantitative comparison with other state-of-the-art low-light enhancement methods to compare the performance of our method. Finally, we perform ablation experiments to analyze and validate the soundness of our network structure.

### 4.1. Dataset and Implementation Details

#### 4.1.1. Datasets

Low-light image enhancement has been a relatively popular research direction, but there are fewer paired-based public datasets in real scenarios. We use publicly available low-light datasets the Brightening Train dataset (Wei et al., 2018) and the LOL dataset (Wei et al., 2018) with a total of 1500 paired images for network training. Website: https://daooshee.github.io/BMVC2018website/. Among them, 1300 are training images, 100 are validation images, and 100 are test images.

During the testing, we randomly selected 50 LOL datasets containing 500 paired images for testing. In addition, we also used the benchmark datasets DICM (Lee et al., 2013), LIME (Guo et al., 2016), MEF (Ma et al., 2015), and NPE (Wang et al., 2013) for testing.

#### 4.1.2. Implementation Details

We use Ubuntu 18.04 operating system with Nvidia GeForce RTX TITAN graphics card. Our network is trained for 300 epochs in the Pytorch deep learning framework. initial learning rate is 0.0001. gradient optimization is performed using the Adam (Kingma and Ba, 2014) optimizer. During the training process, the batch size is set to 4, and the four selected images are randomly cropped to a slice size of 256 × 256 × 3. In addition, for data supplementation, each slice is flipped horizontally with a probability of 0.5.

#### 4.1.3. Evaluation Criteria

In order to be able to evaluate the quality of low-light enhanced images in an all-round way, we use non-referenced evaluation metrics and referenced evaluation metrics to objectively analyze the enhanced images.

The reference-based evaluation metrics requires that the enhanced low-light image is compared with the reference image. We use peak signal-to-noise ratio (PSNR), structural similarity (SSIM) and luminance order error (LOE) to measure the gap between the enhanced image and the ground truth image. PSNR is used to evaluate the image fidelity by calculating the mean square error between the enhanced low-light image and the ground truth image. The higher the value of PSNR, the smaller the image distortion. SSIM considers the image similarity in terms of brightness, contrast and structure, respectively. The larger the value of SSIM, the more similar the structure of the enhanced image is to the ground truth image and the smaller the loss. LOE (Wang et al., 2013) indicates the natural retention ability of the image, and when the value is smaller, the enhanced image is more natural.

Based on the evaluation metrics of no-reference, we used the blind/referenceless image spatial quality evaluator (BRISQUE) (Mittal et al., 2012) and the natural image quality evaluator (NIQE) (Mittal et al., 2013) to evaluate the quality of no-reference images. BRISQUE quantifies the loss of naturalness by using natural scene statistics with locally normalized luminance coefficients. When the BRISQUE value is lower, it indicates less distortion and higher image quality. NIQE is a distance metric between the computed model statistics and the enhanced low-light image based on the spatial domain natural scene statistics model. When the NIQE value is lower, it means that the closer to the natural image, the higher the image quality.

### 4.2. Subjective Evaluation

Our method was compared visually with 10 different state-of-the-art methods under real low-light images. IB (Al-Ameen, 2019) method based on mathematical theory method. FFM (Dai et al., 2019) method based on image fusion. RRM (Li et al., 2018) and SDD (Hao et al., 2020) methods based on Retinex decomposition. MBLLEN (Lv et al., 2018), Retinex-Net (Wei et al., 2018), KinD (Zhang et al., 2019), DLN (Wang et al., 2020a), MIRNet (Zamir et al., 2020), Zero-DCE (Guo et al., 2020) methods based on deep learning.

**Figure 6:**
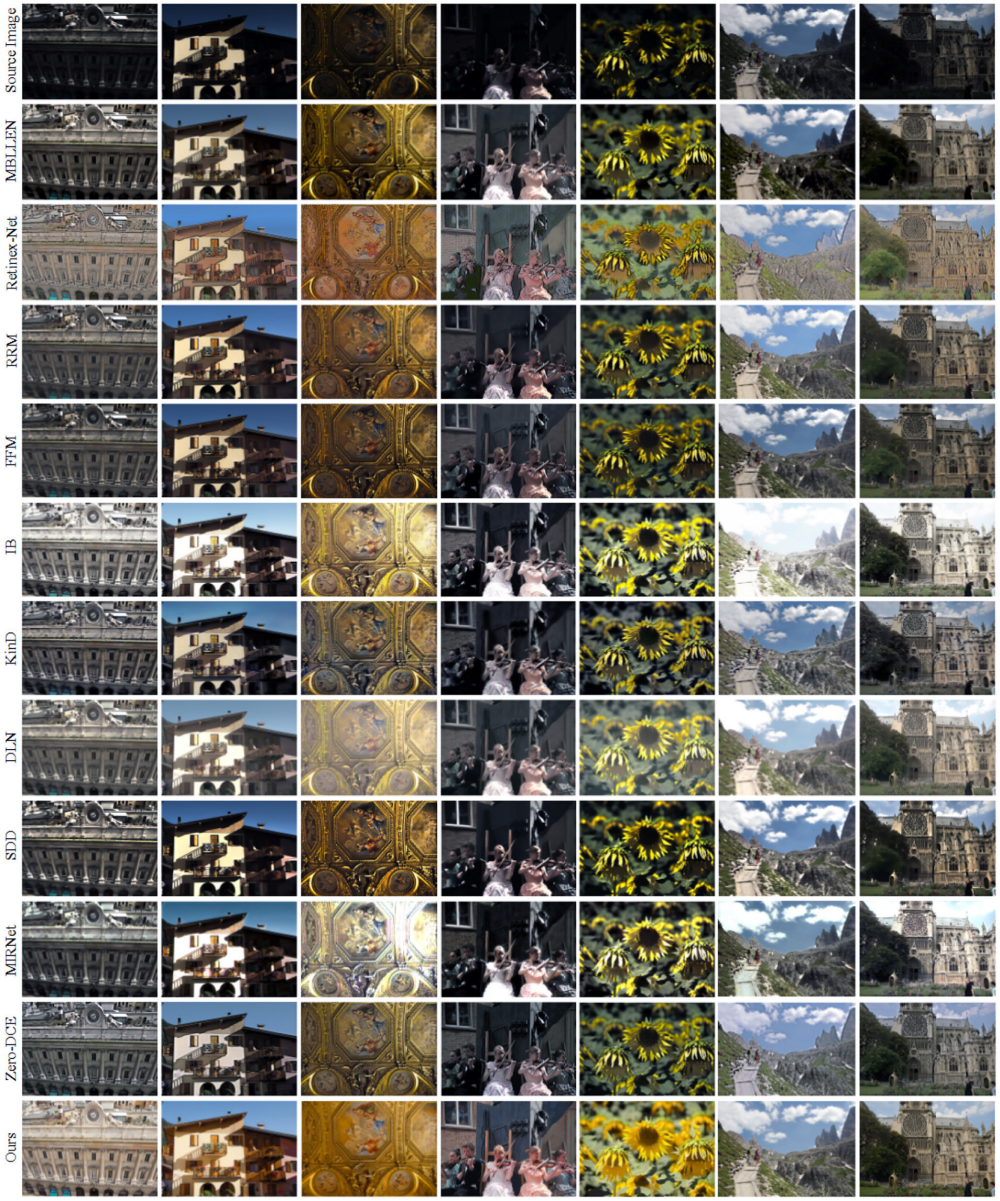
Enhancement results of different low-light enhancement methods in real low-light images. The last row is the enhancement result of our method.

We selected 7 real low-light images for visual comparison of the above 10 low-light enhancement methods. As shown in Figure 6, the first row is the original low-light image, lines 2-11 are the enhancement results of the other 10 comparison methods, and the last row is our enhancement result. It can be seen that the overall brightness of the image is darker after the MBLLEN method with multi-branch shimmer enhancement and the FFM method with fusion frame enhancement for rows 2 and 5, respectively. The Retinex-Net method in row 3 divides the network into a decomposed network and an enhanced network, and the obtained results are heavily sharpened and excessively noisy. RRM and SDD methods based on the Retinex method are shown in rows 4 and 9, respectively, and the enhanced effect suffers from underexposure, with some of the detailed texture features of the image not being recovered. For example, the background area behind the fourth column of the hand violin is missing part of its content. It can be observed from the sixth line that the image enhanced by the IB method has overexposure of brightness and serious color distortion. For example, the sky area of the image in the 6th and 7th columns is over-enhanced, and the details are seriously lost. As seen in rows 8 and 10, the supervised deep learning-based DLN and MIRNet methods suffer from overexposure, poor color saturation, and color distortion. The unsupervised Zero-DCE method has dark colors after enhancement. The last row is our proposed method, and the enhanced image results in uniform color, high contrast, and excellent brightening effect.

**Figure 7:**
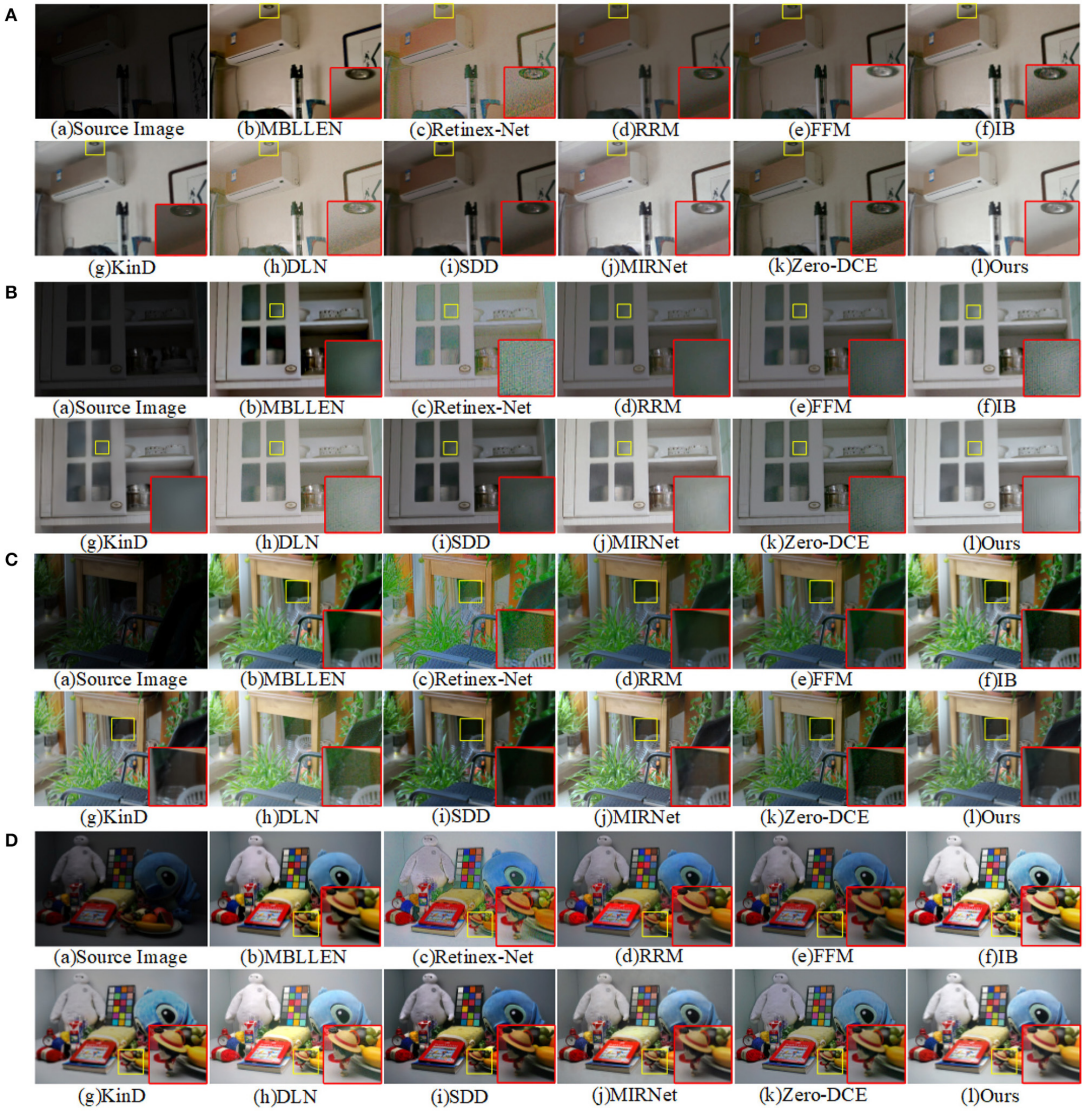
Four images were extracted from the LOL dataset to compare the visual effects of our method with other low-light enhancement methods. **(A)** is the original image, **(B–K)** is the image enhanced by other methods, and **(I)** is the image enhanced by our method. The yellow box is the selected detail content, and the red box is the enlarged detail.

Figure 7 shows the enhancement effects demonstrated by different enhancement methods on the LOL dataset. It can be observed that the overall brightness of the image illumination enhanced by the methods in Figures 7d,e,i,k is insufficient. Although the SDD method in Figure 7i reduces the noise by smoothing, it is still lacking for the brightness enhancement. The enhanced image results of DLN method and MIRNet method have strong noise in the local dark regions. Overall, our proposed method has a good effect on global luminance enhancement, and the enhanced image is colorful and maintains the detailed texture of the image.

**Figure 8:**
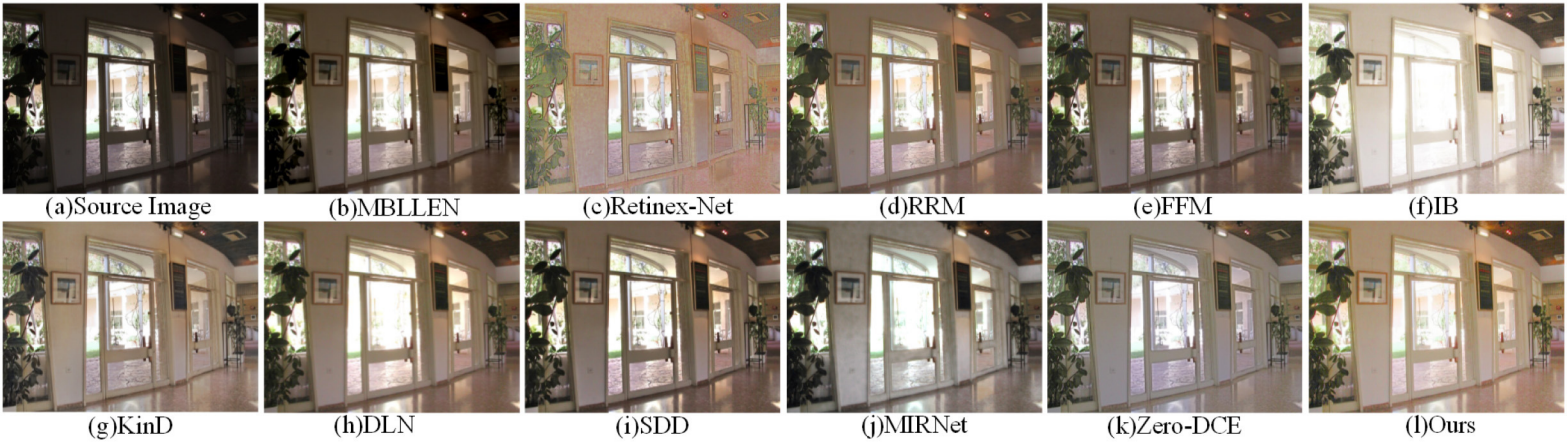
Comparison of the visual effects of our method and other low-light enhancement methods in the DICM dataset. **(A)** is the original image, **(B–K)** is the other 10 low-light enhancement methods, and **(I)** is our method.

Figure 8 shows the enhancement results of the indoor scene map on the DICM dataset. The low-light image after enhancement by the Retinex-Net method is severely sharpened and produces severe noise. The IB method is overexposed and shows partial loss of details. The enhanced result of the MIRNet method shows a large number of color spots and uneven overall brightness adjustment. The overall color reproduction of the enhanced image by our method is high. Thus, our method produced good enhancement results.

**Figure 9:**
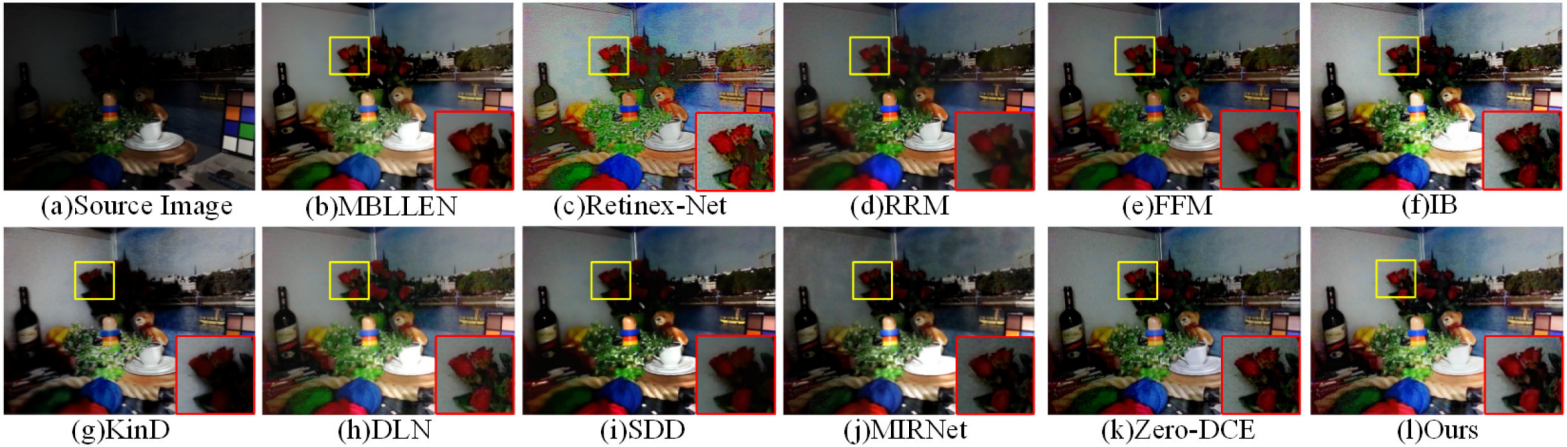
Comparison of the visual effects of our method and other low-light enhancement methods in LIME dataset. The yellow box shows the selected detail content and the red box shows the detail magnification. **(A)** is the original image, **(B–K)** is the other 10 low-light enhancement methods, and **(I)** is our method.

Figure 9 shows the low-light image enhancem-ent in the LIME dataset for an indoor low-light environment. Comparing the enhanced rose color by the detail magnification, the enhancement effect of our method is richer in terms of color. The MBLLEN and RRM methods are smoothed to reduce noise generation, resulting in a severe loss of detail information on the walls. Compared with other enhancement methods, our method achieves better visual results.

**Figure 10:**
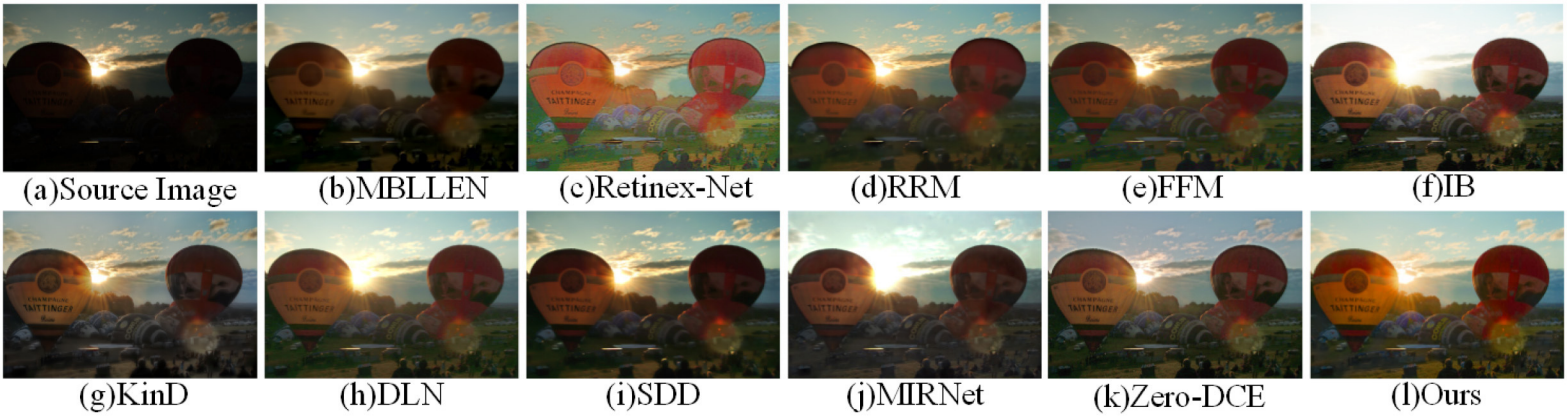
Comparison of the visual effects of our method and other low-light enhancement methods in the MEF dataset. **(A)** is the original image, **(B–K)** is the other 10 low-light enhancement methods, and **(I)** is our method.

Figure 10 shows the low-light image enhancem-ent in the MEF dataset for the outdoor backlit scene. The IB and MIRNet methods are overexposed at the sky, while the KinD method is moderately exposed overall, but distorts the color at the grass. Our method maintains the realism of the sky color, and has good effect on the contrast and brightness enhancement of local details.

**Figure 11:**
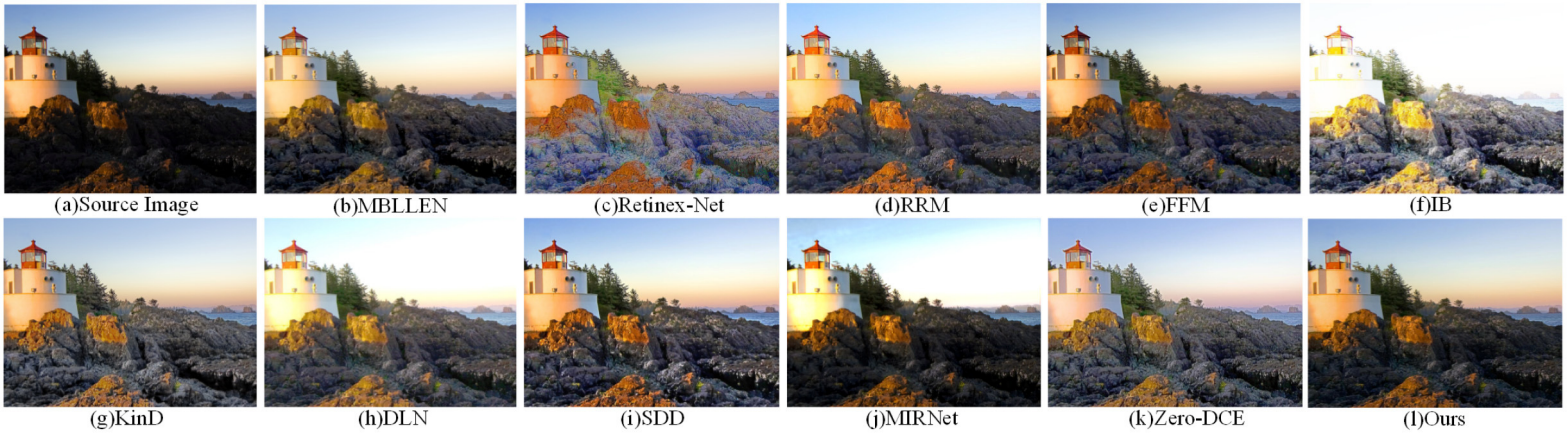
Comparison of the visual effects of our method and other low-light enhancement methods in the NPE dataset. **(A)** is the original image, **(B–K)** is the other 10 low-light enhancement methods, and **(I)** is our method.

Figure 11 shows the effect of low light image enhancement in a natural landscape in the NPE dataset. It can be observed that there is overexposure at the sky for IB, DLN and MIRNet methods. The overexposure of the stone parts in Figures 11c,g,i,k causes severe distortion and lack of naturalness in the image content. In terms of image fidelity and color information, our method is generally superior to other low-light enhancement methods.

### 4.3. Objective Evaluation

To evaluate the performance of our MSFFNet more comprehensively, we analyze various low-light enhancement methods using objective evaluation metrics. As shown in the data in Table 1, we used 11 low-light enhancement methods to enhance the real low-light images in Figure 6. And the performance of the various enhancement methods is evaluated by using PSNR, SSIM, NIQE, BRISQUE and LOE metrics. Table 1 shows that the proposed MSFFNet achieves the best values for various evaluation metrics. It can be seen that our method has some superiority in enhancing detailed textures while maintaining the naturalness of the image.

**Table 1:** Comparison of the real low-light dataset in Figure 6 with the state-of-the-art low-light enhancement methods in metrics PSNR, SSIM, NIQE, BRISQUE, LOE.

**Method**	**PSNR**	**SSIM**	**NIQE**	**BRISQUE**	**LOE**
MBLLEN	17.246	0.675	4.472	28.661	664.876
Retinex-Net	19.251	0.729	5.612	38.42	687.721
RRM	18.496	0.686	5.992	35.388	665.23
FFM	16.131	0.704	4.33	26.238	669.866
IB	18.773	0.799	4.886	26.035	661.516
KinD	18.455	0.728	4.651	24.71	666.975
DLN	20.454	0.826	4.261	22.978	664.835
SDD	17.576	0.672	5.203	29.382	674.554
MIRNet	16.718	0.695	4.427	27.87	676.466
Zero-DCE	19.555	0.795	4.456	24.734	663.421
Ours	**25.879**	**0.878**	**4.193**	**22.475**	**659.311**

*The best results are marked in bold red*.

In addition, for the reference LOL dataset, we have also quantitatively evaluated various low-light enhancement methods using various objective evaluation metrics. As shown in the data in Table 2, comparing various state-of-the-art low-light enhancement methods, our method has superior results in smoothing noise and restoring image naturalness. In addition, the MIRNet method also shows better results under most other metrics. As shown in Figure 7, our method can recover more texture information in a visual perspective, preserving the naturalness of the original image.

**Table 2:** Quantitative comparison with state-of-the-art methods on the LOL dataset.

**Method**	**PSNR**	**SSIM**	**NIQE**	**BRISQUE**	**LOE**
MBLLEN	16.477	0.773	4.954	26.891	576.325
Retinex-Net	16.54	0.55	8.528	29.964	650.367
RRM	12.256	0.728	5.703	31.644	620.891
FFM	11.541	0.63	6.435	19.209	625.354
IB	20.203	0.739	6.97	21.895	582.239
KinD	18.254	0.866	5.671	25.7	638.234
DLN	21.03	0.812	5.613	18.566	583.655
SDD	11.957	0.707	5.125	25.532	613.574
MIRNet	26.041	0.912	4.573	29.185	556.238
Zero-DCE	14.291	0.717	6.881	22.216	598.564
Ours	**28.396**	**0.935**	**4.341**	**16.536**	**547.325**

*The best results are indicated in bold red*.

The four non-referenced data sets of DICM, LIME, MEF, and NPE were quantitatively compared using NIQE and BRISQUE non-referenced quality evaluation indicators, respect-ively. The image quality recovered by various enhancement methods was evaluated by spatial quality assessment and naturalness of the enhanced images. The statistical results in Table 3 show that our method outperforms various advanced low-light enhancement metho-ds in all metric values, proving equally good performance in the no-reference dataset. For aspects such as image fidelity and spatial detail, which are of interest for evaluation metrics, our method has a great advantage.

**Table 3:** Performance of the following low-light enhancement methods calculated on the DICM, LIME, MEF, and NPE public datasets using the no-reference metrics NIQE and BRISQUE.

	**NIQE**				**BRISQUE**			
**Method**	**DICM**	**LIME**	**MEF**	**NPE**	**DICM**	**LIME**	**MEF**	**NPE**
MBLLEN	3.707	4.427	4.549	3.902	24.96	26.089	31.807	28.348
Retinex-Net	4.488	4.869	4.41	4.461	31.289	32.088	20.607	30.537
RRM	3.953	4.902	4.918	4.591	32.044	32.07	32.644	33.328
FFM	3.23	3.99	3.665	3.46	22.117	18.388	18.204	23.05
IB	3.211	4.191	3.735	3.961	26.909	22.496	17.895	33.871
KinD	3.564	4.628	3.877	3.443	25.238	23.681	27.505	23.336
DLN	3.009	4.054	3.356	3.571	20.19	18.656	17.165	25.754
SDD	3.554	4.302	4.268	3.54	28.267	24.163	28.095	26.238
MIRNet	3.133	4.008	3.77	3.701	17.926	21.634	25.275	23.431
Zero-DCE	2.899	3.997	3.697	3.527	19.215	19.473	16.604	26.581
Ours	**2.847**	**3.938**	**3.291**	**3.422**	**17.228**	**17.74**	**14.243**	**22.492**

*The best results are marked in bold red*.

**Figure 12:**
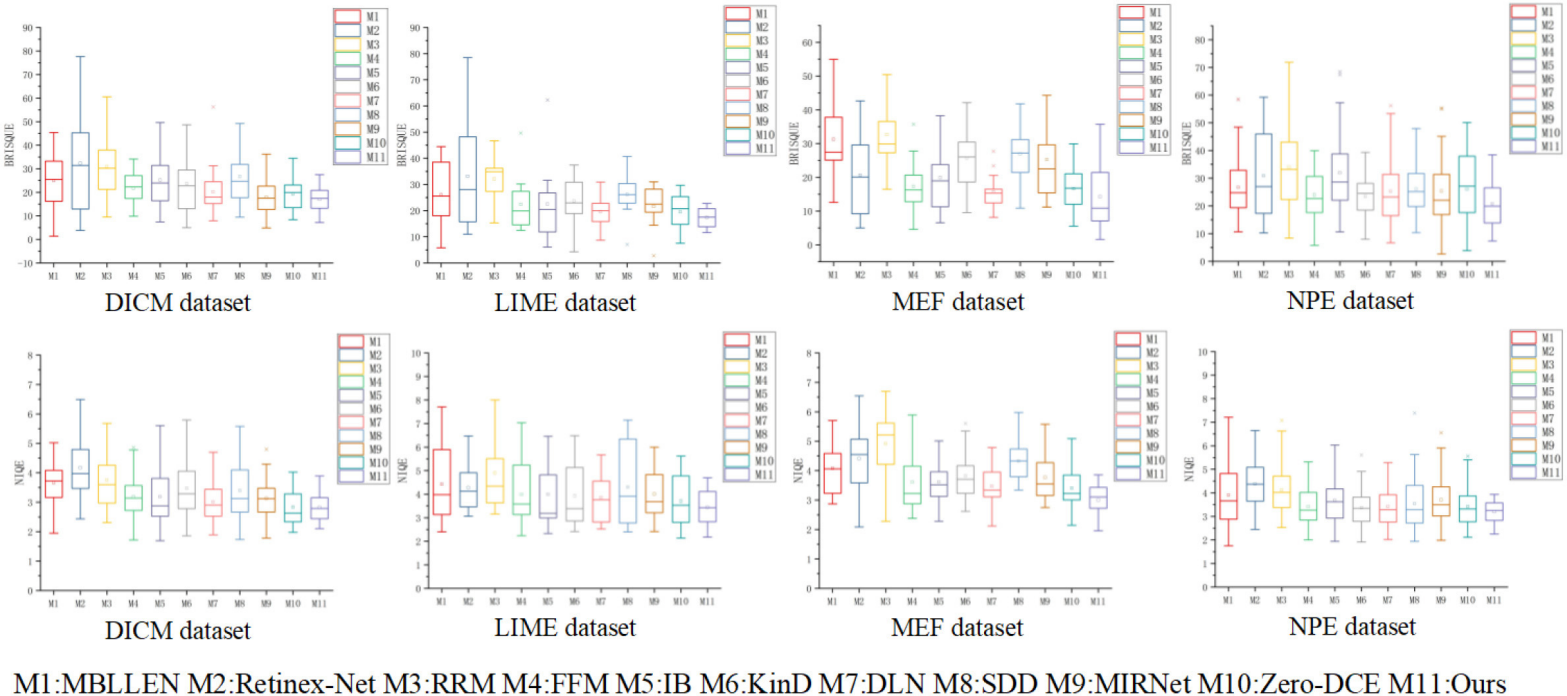
The results of the different low-light enhancement methods were calculated using BRISQUE and NIQE evaluation metrics in a total of four public datasets, DICM, LIME, MEF and NPE. The calculated results are compared quantitatively using box-line plots. The shorter the length of the box line plot, the more stable the method is and the better the enhancement effect.

In Figure 12, we present the numerical results of the BRISQUE and NIQE evaluation metrics using box line plots for statistics to visualize the distribution of metric values for various low-light enhancement methods. The first row shows the result of BRISQUE score statistics. The second row shows the results of the fraction statistics of NIQE. It is observed in Figure 12 that the Retinex-Net method has a larger range of fluctuation in values on BRISQUE, indicating that the enhanced images are weaker in spatial domain quality than the other low-light enhancement methods. Our method achieves low and concentrated values on BRISQUE and NIQE, indicating that the enhanced images are more naturalistic and fidelity.

### 4.4. User Study

Since existing publicly available low-light image datasets lack images in normal light for comparison, it is impossible to judge the difference between the enhanced results and ground truth images. The effect of low-light image enhancement can rely on people's subjective judgment to rate the image quality. Therefore, we conducted a user study through an observational method to evaluate the performance of various low-light image enhancement methods based on users' subjective judgments.

First, we randomly selected 10 low-light images in 4 public datasets (DICM, LIME, MEF, NPE) and performed image enhancement using MBLLEN, Retinex-Net, RRM, FFM, IB, KinD, DLN, SDD, MIRNet, Zero-DCE, and our method. Second, we invited 20 participants (10 males and 10 females) with normal vision to subjectively evaluate the results of the low-light image enhancement methods. The participants compared the low-light image with the enhanced image to see how well the image was recovered. If the participant thought the enhancement was good, the image was scored 1; otherwise, it was scored 0. All image evaluations were performed under a monitor with a resolution of 1,920*1,080. The scoring details were as follows: 1) Whether there was a lot of noise and texture loss in the enhanced image. 2) Whether there was color imbalance and uneven color in the enhanced image. 3) Whether there was overexposed or underexposed in the enhanced image.

After counting the participant assessment scores, the ranking results were derived by estimating subjective scores using the Bradley-Terry model (Bradley and Terry, 1952). In addition, we calculated the sum, mean, standard deviation and variance statistics of the scores of the different enhancement methods separately. The final results are shown in Table 4. Combining the numerical results of several metrics, our methods were more favored for human visual observation. Among them, SDD and DLN methods also achieved excellent ranking in the quality of enhancement effects. SDD method uses an efficient semi-decoupled approach for Retinex decomposition, achieving efficient visibility and image quality. DLN method used the inverse projection method iteratively for residual learning and achieved good results in the processing of noise and texture.

**Table 4:** Subjective evaluation scores of the enhancement results of 10 low-light images by 20 participants under 11 low-light enhancement methods.

**Method**	**Sum↑**	**Mean↑**	**Variance↓**	**Standard deviation↓**	**Rank↓**
MBLLEN	151	15.1	0.767	0.876	5
Retinex-Net	136	13.6	0.489	0.699	11
RRM	144	14.4	0.711	0.843	7
FFM	148	14.8	1.067	1.033	6
IB	137	13.7	1.344	1.16	9
KinD	141	14.1	1.211	1.101	8
DLN	153	15.3	2.233	1.494	3
SDD	158	15.8	0.622	0.789	2
MIRNet	138	13.8	0.9	0.949	10
Zero-DCE	152	15.2	1.733	1.317	4
Ours	173	17.3	0.456	0.675	1

### 4.5. Ablation Study

**Figure 13:**
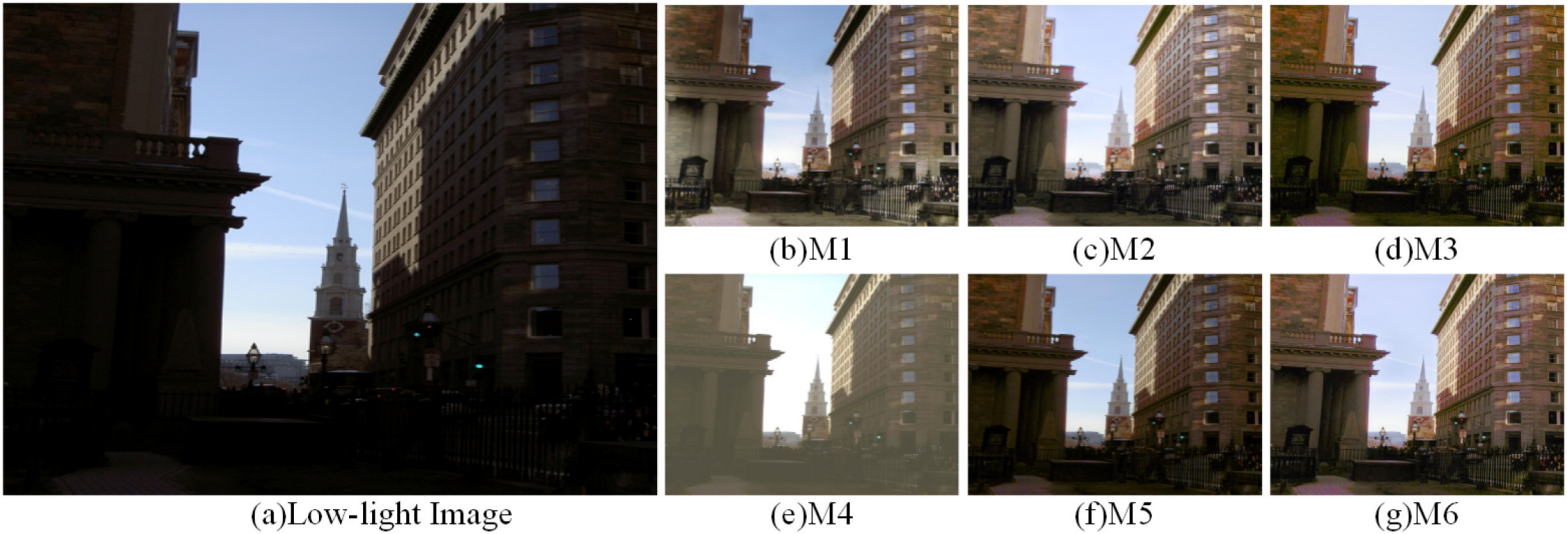
Visual comparison results with different combinations of modules in the DICM dataset. **(A)** shows the low light image and **(B–G)** show the effect of the enhanced low light image with different combinations of modules.

**Figure 14:**
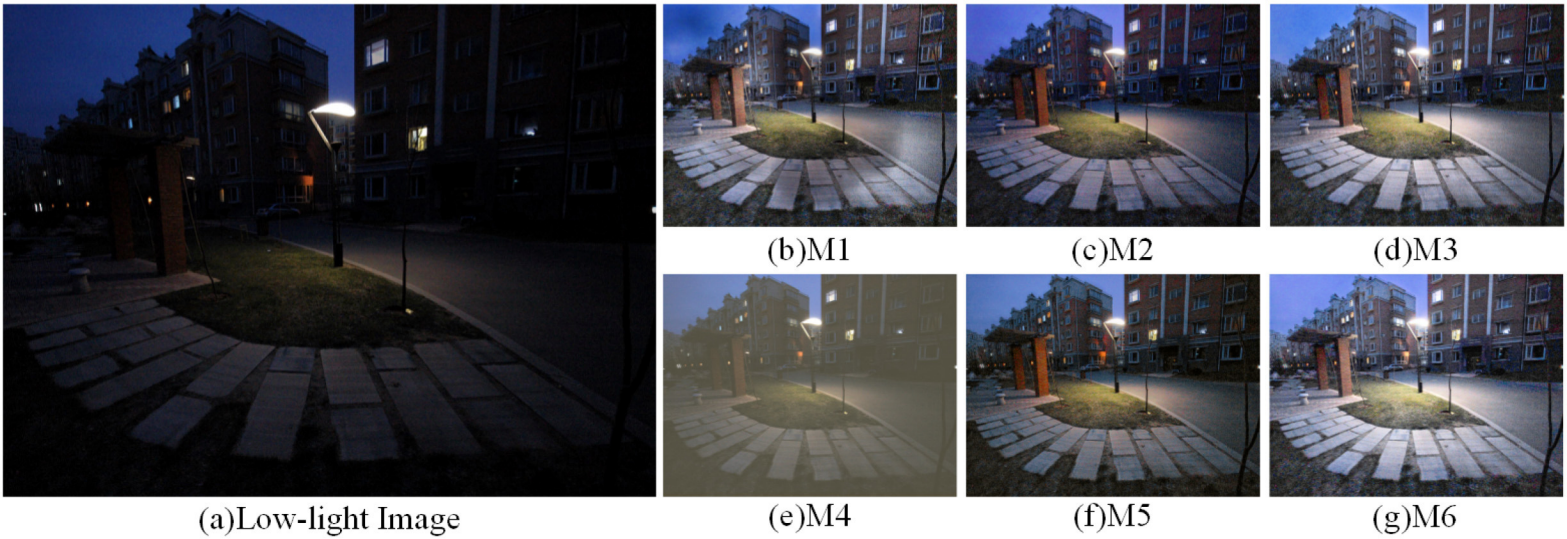
Visual comparison results with different combinations of modules in the LIME dataset. **(A)** shows the low light image and **(B–G)** show the effect of the enhanced low light image with different combinations of modules.

**Figure 15:**
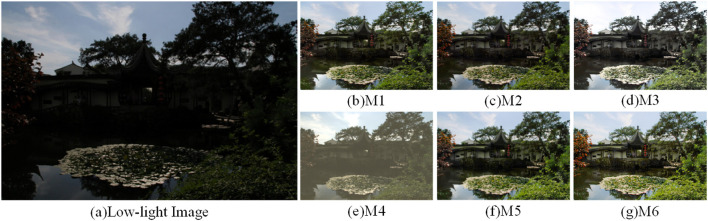
Visual comparison results with different combinations of modules in the MEF dataset. **(A)** shows the low light image and **(B–G)** show the effect of the enhanced low light image with different combinations of modules.

To verify the performance of each module, six ablation experiments were performed to determine the importance and rationality of adding modules. It can be observed that adding all modules gives better performance than eliminating any of them. Methods M1-M6 are represented as the combination modes of each module, as shown in Table 5. Figures 13–15 shows the qualitative comparison results of different module combinations under various common data sets. In the ablation experiment, M1-M6 methods kept the training set, learning rate and training times unchanged.

**Table 5:** Validation of the rationality of the proposed network structure by adding and removing different modules in the ablation experiments.

**Model**	**CBAM**	**AFM**	**FCM**	**CFM**	**MIMO**
M1				✓	✓
M2	✓	✓		✓	✓
M3			✓	✓	✓
M4	✓	✓	✓	✓	
M5	✓	✓	✓		✓
M6	✓	✓	✓	✓	✓

*Notice that we represent the multiscale input and multiscale output images as MIMO*.

For the M1 method, which lacks the attention mechanism and FCM, the image enhancement effect in Figures 13–15b shows severe color imbalance and uneven brightness enhancement. In Figure 13b, the buildings are partially white and the grass color is also degraded to different degrees, and the enhanced image loses its original color. Figure 14b The enhanced image has local dark patches and the brightness of the enhanced image is unbalanced. Figure 15b The details below the pavilion are blurred, and the left half of the lake shows color distortion.

For the M2, M3, and M5 methods, which lack the network structure of FCM, attention mechanism and CFM, respectively, the presented results all have strong noise and low exposure. Figures 13c,f–15c,f the whole image exhibits a low exposure state. Figure 15d lacks the attention mechanism, resulting in an overall color imbalance and overexposure in the sky.

For the M4 method remove the multiscale input and output images to verify the effectiveness of our multiscale network structure. It can be clearly seen that the image enhancement results in Figures 13–15e have poor quality. Overall, the comparative analysis of the above ablation experiments concludes that the M6 method with all modules added outperforms the results of adding some modules in terms of contrast, color balance, and detailed texture recovery. Therefore, the designed network structure is reasonable and scientific.

## 5. Conclusion

In this paper, we propose an Attention-Guided Multi-scale feature fusion network (MSFFNet) for low-light image enhancement to solve the low-light image enhancement problem. To avoid the computational burden caused by stacking multiple sub-networks, a simple and efficient single encoder-decoder structure is used for low-light image enhancement in a coarse-to-fine strategy. The original encoder structure is changed to a multi-scale input low-light image and combined with feature information at different scales. It also combines CBAM, which can accurately focus on the dark region and strengthen the network's ability to extract hidden feature information. The decoder structure is changed to multi-scale output low-light-enhanced images for multi-scale frequency domain loss calculation, which effectively supervises the recovery of low-light-enhanced images in the image reconstruction process. FCM enhances the channel and spatial dependence in the feature map and reduces the loss of details. The CFM is introduced to fuse semantic information at different scales, and fully combines shallow semantic information and deep spatial information for dynamic low-light image enhancement. Attention fusion strategy is adopted to introduce AFM to extract attentional features from different layers, and fully fuse the detailed texture features of the low-level feature maps and the semantic information of the high-level feature maps. Finally, after extensive experiments, MSFFNet has been shown to be more effective than other methods in terms of visual effects and metric scores. In the next work, we will continue to investigate more efficient low-light enhancement methods to improve the robustness and generalization ability of the network.

## Data Availability Statement

The raw data supporting the conclusions of this article will be made available by the authors, without undue reservation.

## Author Contributions

All authors listed have made a substantial, direct, and intellectual contribution to the work and approved it for publication.

## Funding

This research was supported by the National Natural Science Foundation of China (61772319, 62002200, 12001327, and 62176140) and Shandong Natural Science Foundation of China (ZR2020QF012 and ZR2021MF068).

## Conflict of Interest

The authors declare that the research was conducted in the absence of any commercial or financial relationships that could be construed as a potential conflict of interest.

## Publisher's Note

All claims expressed in this article are solely those of the authors and do not necessarily represent those of their affiliated organizations, or those of the publisher, the editors and the reviewers. Any product that may be evaluated in this article, or claim that may be made by its manufacturer, is not guaranteed or endorsed by the publisher.
